# Efficacy and safety profile of neuroendoscopic hematoma evacuation combined with intraventricular lavage in severe intraventricular hemorrhage patients

**DOI:** 10.1002/brb3.1756

**Published:** 2020-08-18

**Authors:** Hai‐Tao Ding, Yao Han, De‐Ke Sun, Quan‐Min Nie

**Affiliations:** ^1^ Department of Neurosurgery Linyi Central Hospital Linyi Shandong Province China; ^2^ Department of Neurosurgery Weifang People's Hospital Weifang Shandong Province China

**Keywords:** intraventricular lavage, neuroendoscopic hematoma evacuation, severe intraventricular hemorrhage

## Abstract

**Objective:**

The present study was conducted to explore the effect of neuroendoscopic hematoma evacuation in severe intraventricular hemorrhage (IVH).

**Methods:**

Totally 81 patients with severe IVH in our hospital from November 2017 to March 2019 were divided into the intervention group (38 cases who received neuroendoscopic hematoma evacuation combined with intraventricular lavage) and the control group (40 cases who received trepanation drainage). The perioperative condition, hematoma clearance rate, Glasgow coma score (GCS), hematoma recurrence rate, and prognosis were observed and compared between the two groups after treatment.

**Results:**

The operative time, time of cerebrospinal fluid drainage, and intracranial infection rate in the intervention group elicited superior results to those in the control group (*p* < .05). The clearance rate of hematoma in the intervention group was higher than that in the control group at 6 hr, 1, 3, and 7 days postoperatively (*p* < .05). The postoperative 3‐ and 7‐day GCS scores in the intervention group were higher than those in the control group, and the recurrence rate of hematoma in the intervention group was significantly lower than that in the control group (*p* < .05), and the good/excellent rate of ADL in the intervention group was significantly higher than that in the control group (*p* < .05).

**Conclusion:**

Neuroendoscopic hematoma evacuation combined with intraventricular lavage showed evident beneficial outcomes in patients with severe IVH. It can effectively improve the perioperative condition and improve the hematoma clearance rate and is beneficial to the prognosis of patients with severe IVH.

## 1 INTRODUCTION

Intraventricular hemorrhage (IVH) is one of the common severe conditions in neurosurgery with an acute onset, rapid progress, high disability, and mortality. Especially in cases of severe IVH, hypothalamus and brainstem symptoms will appear first, and thalamic hemorrhage is the most serious cerebral hemorrhage, accompanied by a variety of complications with a high disability and mortality rate (Clavier et al., 2018; Fu, Ng, & Chua, 2019). The most important part to treat patients with severe IVH is to remove IVH as soon as possible, dredge cerebrospinal fluid (CSF) circulation, prevent intracranial hypertension, and minimize secondary brain damage. In the traditional treatment, extraventricular drainage can dredge the CSF circulation and reduce the intracranial pressure, but with a high incidence of rebleeding and intracranial infection (Ge et al., 2019; Masoom Abbas, Gopal Varma, Sankar, & Pai, 2019). Therefore, it is always necessary to find a safe and effective treatment for patients with severe IVH in order to quickly eliminate ventricular hemorrhage and prevent recurrent bleeding. The current study aims to investigate the efficacy and safety of neuroendoscopic hematoma evacuation combined with intraventricular lavage in patients with severe IVH.

## 2 MATERIALS AND METHODS

### 2.1 Participants

A total of 81 patients with severe IVH at our hospital from November 2017 to March 2019 were divided into the intervention group (*n* = 38) and the control group (*n* = 40). In the intervention group, there were 26 males and 12 females, aged from 20 to 65 years, with an average age of (45.78 ± 7.83) years. There were 28 cases of secondary IVH and 10 cases of primary ventricular hemorrhage. The sites of hemorrhage included the head of the caudate nucleus (*n* = 5), thalamus (*n* = 11), and basal nucleus (*n* = 22). In the control group, there were 29 males and 11 females, aged 21–64 years, with an average age of (45.08 ± 8.01) years, and 31 cases had secondary IVH and 9 cases had primary ventricular hemorrhage. The sites of hemorrhage included the head of the caudate nucleus (*n* = 4), thalamus (*n* = 11), and basal nucleus (*n* = 25). There was no significant difference in age and other general data between the two groups (*p* > .05). The general information of the two groups at the time of admission is shown in Table 1.

**Table 1:** General data of two groups of patients

Parameter	Intervention group (*n* = 38)	Control group (*n* = 40)	*x* ^2^/*t*	*p* Value
Gender (case, %)				
Male	26	29	1.823	>.05
Female	12	11		>.05
Age ($\bar{x}\pm s$, year)	45.78 ± 7.83	45.08 ± 8.01	1.972	>.05
Admission score ($\bar{x}\pm s$, point)				
Course of the disease ($\bar{x}\pm s$, hr)	5.64 ± 1.04	5.77 ± 1.03	2.515	.218
GCS ($\bar{x}\pm s$, score)	10.63 ± 1.88	10.73 ± 1.46	3.189	.096
Greab score	9.67 ± 2.37	9.41 ± 1.68	1.732	.087
Hypertension (case, %)	15 (39.40)	14 (41.42)	2.632	.119
Coronary artery disease (case, %)	10 (30.30)	9 (27.27)	2.765	.083
Hyperlipidemia (case, %)	8 (24.24)	7 (21.21)	3.124	.145
Site of bleeding (case, %)				
Secondary intraventricular hemorrhage	28 (73.68)	31 (77.50)	1.542	.098
Primary intraventricular hemorrhage	10 (26.32)	9 (22.50)	1.367	.11
Head of caudate nucleus	5 (33.26)	4 (39.42)	3.815	.084
Thalamus	11 (21.24)	11 (22.27)	3.565	.253
Basal nucleus	22 (35.24)	25 (38.41)	4.154	.093
Bleeding volume ($\bar{x}\pm s$, ml)	23.26 ± 1.24	24.65 ± 1.39	1.324	.081
Hematoma volume ($\bar{x}\pm s$, ml)	48.5 ± 8.7	52.7 ± 9.3	4.152	.162
Surgical data				
Time of onset to surgery ($\bar{x}\pm s$, hr)	2.79 ± 1.42	2.98 ± 0.91	1.292	.210
Hydrocephalus (case, %)	3 (7.89)	17 (42.50)	1.192	.091

To be included in the current study, patients should meet the following inclusion criteria: (a) patients were diagnosed based on the diagnostic criteria of moderate‐to‐severe cerebral hemorrhage in China Guidelines for Diagnosis and Treatment of Cerebral Hemorrhage in 2014 (Branch, 2015); (b) patients with stable vital signs and a GCS score >10 at admission; (c) patients were diagnosed with secondary periventricular hemorrhage into the ventricle or spontaneous ventricular hemorrhage by imaging examination, and the volume of cerebral parenchyma hemorrhage was <30 ml; (d) the experiment of our study was approved by the Ethics Committee and informed consent was provided by the family members of the patients; (e) patients with complete clinical data; (f) the Graeb score of IVH was ≥8. The exclusion criteria were as follows: (a) a previous history of cerebral infarction; (b) hemorrhage of intracranial aneurysms and arteriovenous malformations, craniocerebral trauma, and brain tumor complicated with hemorrhage; (c) incomplete clinical data; (d) abnormal coagulation function; (e) important organ dysfunction; (f) pregnant or lactating women; (g) there were indications for bone flap removal for decompression.

### 2.2 Surgical methods

#### 2.2.1 The intervention group

The intervention group underwent neuroendoscopic hematoma evacuation combined with intraventricular lavage. After general anesthesia, the pathway into the ventricle was determined through the triangular cortex and/or forehead according to the location and size of the hematoma. An arc incision of 5 cm was made in the scalp, about the diameter of the skull window (3 cm), the dura mater was cut and open. Following cauterization of the cortex, the transparent drainage tube with tube core was inserted, and the inner core was removed to facilitate the outflow of hematoma and bloody CSF. The 30° and 0° neuroendoscope and monitoring system by Storz Co. was used to remove intraventricular hematoma with the application of neuroendoscope and its monitoring system. The depth and angle of the drainage tube under the neuroendoscope was adjusted in order to completely remove the hematoma in other parts. After clearance, ventricular lavage was repeatedly performed for about 25 min with a volume of 500 ml. Gentamicin (40,000 units) combined with 500 ml saline at 37°C was used in the lavage fluid. If the patient had a secondary IVH, the angle and depth of the drainage tube was adjusted with the application of the neuroendoscope to clean hemorrhage from the thalamus and basal nucleus. If the patient had a cast of the fourth ventricle, a median incision was made to expose the occipital bone, and the diameter of the milled bone window was about 3 cm. The dura mater was cut and the cortex was electrocauterized, which was located in the nonvascularized area on the surface of the brain, and the drainage tube was placed into the fourth ventricle. Hematoma in the fourth ventricle was removed with the application of the neuroendoscope.

#### 2.2.2 The control group

The control group was given trepanation drainage intervention, with the midline side opening (2.5 cm) as the frontal drilling point, and an incision was made in the scalp 1 cm anterior to the coronal suture, and the dura mater was penetrated by electric drilling. A drainage tube was placed in the ventricle of the brain. After hematoma and bloody CSF flowed out, the drainage device was connected and fixed.

Six hours postoperatively, the patients in the two groups were re‐examined by cranial CT. According to the results of cranial CT, if there was no further bleeding, residual hematoma could be drained by the lumbar cistern according to the drainage of CSF.

### 2.3 Observation index

The perioperative condition, CSF drainage, hematoma clearance rate, GCS score, hematoma recurrence rate, and prognosis were observed and compared between the two groups after treatment.

Perioperative condition: including operative time, CSF drainage time, intracranial infection rate and so on.

GCS score: The GCS scores at 3 and 7 days postoperatively were recorded, respectively.

Hematoma clearance rate: The hematoma clearance rates at 6 hr, 1 day, 3 days, and 7 days after surgery were recorded and compared. Hematoma clearance rate = (preoperative intracerebral hematoma volume − postoperative intracerebral hematoma volume)/preoperative hematoma volume. The volume of intracranial hematoma was calculated using the Toda formula according to the results of the patient's preoperative cranial CT. All patients were routinely reviewed by cranial CT after surgery, and the volume of residual intracranial hematoma was calculated according to cranial CT.

Prognosis: Activities of daily living (ADL) (Mlinac & Feng, 2016) were used to evaluate the prognosis of the patients 3 months postoperatively, which was divided into five grades, including Grade V: the patients were in the vegetative state or died; Grade IV: patients were conscious but need to be taken care of, and stay in bed for a long time; Grade cIII: patients need help in walking and living; Grade II: patients can live independently or some of their functions can recover; Grade I: patients can return to normal completely and can live a normal life. The poor effect was Grade IV–V, and the good effect was Grade I–III.

### 2.4 Statistical methods

Statistical analysis was carried out by SPSS 18.0 software, and data were expressed by ($\bar{x}\pm s$) and *t* test. The number and percentage of categorical data were analyzed by chi‐squared test or Fisher accurate test. A *p* < .05 represented statistically significant difference.

## 3 RESULTS

### 3.1 Comparison of clinical data between the two groups

The two groups were comparable in age, gender, and other parameters between the two groups (*p* > .05) (Table 1).

### 3.2 Comparison of perioperative data between the two groups

The operative time, CSF drainage time, and intracranial infection rate in the intervention group were superior to those in the control group (*p* < .05) (Table 2).

**Table 2:** Comparison of perioperative data between the two groups

Group	Operative time (hr)	Cerebrospinal fluid drainage time (hr)	Intracranial infection rate (%)
Intervention group (*n* = 38)	3.04 ± 0.62	4.32 ± 0.73	1 (2.63)
Control group (*n* = 40)	3.27 ± 0.71	7.74 ± 0.98	8 (20.00)
Test value	9.632^b^	12.93^b^	11.83^a^
*p* value	<.01	<.01	<.01

Note

^a^represented for *x* ^2^ values and ^b^for *t* values.

### 3.3 Comparison of hematoma clearance rate between the two groups

The clearance rate of hematoma in the intervention group was higher than that in the control group at 6 hr, 1 day, 3 days, and 7 days postoperatively (*p* < .05) (Table 3).

**Table 3:** Comparison of hematoma clearance rate between the two groups (%)

Group	6 hr postoperatively	1 day postoperatively	3 days postoperatively	7 days postoperatively
Intervention group (*n* = 38)	6 (15.79)	27 (71.05)	33 (86.84)	34 (89.47)
Control group (*n* = 40)	2 (5.00)	16 (40.00)	26 (65.00)	31 (77.50)
*x* ^2^ value	13.082	9.121	12.67	11.01
*p* Value	<.01	<.01	<.01	<.01

### 3.4 Comparison of postoperative 3‐ and 7‐day GCS score and recurrence rate of hematoma between the two groups

Postoperatively, the scores of 3‐ and 7‐day GCS in the intervention group were higher than those in the control group, and the recurrence rate of hematoma in the intervention group was significantly lower than that in the control group (Table 4).

**Table 4:** Comparison of postoperative 3‐ and 7‐day GCS scores and recurrence rate of hematoma between the two groups

Group	Postoperative 3‐day GCS score (point)	Postoperative 7‐day GCS score	Recurrence rate of hematoma (%)
Intervention group (*n* = 38)	11.08 ± 2.61^b^	12.78 ± 2.41^b^	1 (2.63)^a^
Control group (*n* = 40)	7.03 ± 2.02	8.93 ± 2.71	4 (7.50)
Text value	15.839	19.832	16.221
*p* Value	<.01	<.01	<.01

Note

^a^represented for *x* ^2^ values and ^b^for *t* values.

### 3.5 Comparison of ADL between the two groups

The good/excellent rate of ADL in the intervention group was significantly higher than that in the control group (*p* < .05) (Table 5).

**Table 5:** Comparison of ADL between two groups (%)

Group	I	II	III	IV	V	Good/excellence rate
Intervention group (*n* = 38)	7 (18.42)	16 (42.11)	12 (31.58)	2 (5.26)	1 (2.63)	35 (92.11)
Control group (*n* = 40)	2 (5.00)	14 (35.00)	17 (42.50)	4 (10.00)	3 (7.50)	33 (82.50)
*x* ^2^ value						14.091
*p* Value						<.01

### 3.6 Analysis of CSF drainage

The CSF drainage volume at 24 hr, 3 days, and 7 days postoperatively was recorded (Table 6). The average drainage volume of CSF in the intervention group was significantly higher than that in the control group 24 hr and 3 days postoperatively, but the drainage volume at day 7 was smaller than that in the control group (*p* < .05), indicating that the CSF in the intervention group could be fully drained in a short time.

**Table 6:** CSF drainage in both groups

	24 hr Cerebrospinal fluid drainage (ml $\bar{x}\pm s$)	3 days Cerebrospinal fluid drainage (ml $\bar{x}\pm s$)	7 days Cerebrospinal fluid drainage (ml $\bar{x}\pm s$)
Intervention group (*n* = 38)	300.5 ± 24.0	245.5 ± 39.0	119.0 ± 34.5
Control group (*n* = 40)	100.5 ± 15.0	125.6 ± 23.6	245.5 ± 22.1
Text value	8.456	12.231	7.845
*p* Value	<.01	<.01	<.01

## 4 DISCUSSION

The deep part of the brain is the site of IVH, which can be divided into secondary and primary according to the type of hemorrhage. Combined with craniocerebral CT, it can be divided into deep nucleus hemorrhage and ventricular hemorrhage, such as the caudate nucleus head, basal nucleus, and thalamus. IVH has a negative effect on CSF circulation, resulting in high intracranial pressure and compression of the brainstem and other symptoms. The typical clinical symptoms of patients with severe IVH include the disturbance of water and electrolyte balance, myotonia, limb dysfunction, and disturbance of consciousness with a high disability and mortality rate (Nakashima, Iijima, Muraoka, & Koketsu, 2019). Epidemiological studies (Abate et al., 2019) suggest that the mortality associated with IVH is as high as 80%. The main reasons for the poor prognosis of severe IVH are as follows: (a) severe IVH can lead to high intracranial pressure; at the same time, it can activate the expression of inflammatory factors in the brain tissue, decompose hemoglobin, and produce free radicals in the process of hematoma absorption. It can be combined with vasospasm and has a serious effect on brain function and metabolism of brain tissue (Amuluru, Al‐Mufti, Romero, & Gandhi, 2018). (b) Secondary or primary IVH can lead to the compression of the third and fourth ventricles, resulting in obstructive hydrocephalus, and ventricular casting can occur in severe cases. It can even lead to the occurrence of central cerebral hernia, which is closely related to acute obstruction of the CSF circulation pathway (Yamaguchi, Kohga, Tosaka, Yoshimoto, & Ishihara, 2015). (c) Hematoma and bloody CSF can affect hypothalamic function and lead to a series of serious complications such as water and electrolyte disturbance. In the long term, it can cause “cobweb” ventricle, and in serious cases, it can lead to communicating hydrocephalus, which is mainly due to the disturbance of CSF circulation (Wang et al., 2016). Clearly, the early removal of hematoma to reduce high intracranial pressure and the regulate CSF circulation are considered the most important part in terms of treating severe IVH.

The main intervention of severe IVH is surgery, which can be divided into neuroendoscopic surgery combined with intraventricular lavage and routine trepanation and drainage. Among them, ventriculocentesis is regarded as a routine trepanation and drainage, which is simple and convenient and widely used in primary care hospitals and is the main approach of clinical treatment of the disease. This operation has therapeutic effect on patients with the disease, but due to the influence of blood coagulation, drainage tube side hole and other factors, some patients will have postoperative drainage tube blockage. The compression by hematoma and the obstruction of the ventricular system cannot be relieved in a short time. Although urokinase can relieve the compression of hematoma and the obstruction of the ventricular system, it can lead to new bleeding, and repeated uses of urokinase will increase the risk of new bleeding and intracranial infection. With continuous progress of medical science and technology, neuroendoscopic technology has also made excellent progress. According to several scholars (Du et al., 2018; Haldar & Singh Bajwa, 2019; Song et al., 2018), the neuroendoscope can effectively remove intracranial hematoma and has the advantages of less trauma and simple operation, which is a new method for treatment of cerebral hemorrhage. In recent years, the application of neuroendoscopes in the treatment of IVH has also been developed. This experiment provides a basis for the clinical application of neuroendoscopes in intracerebral hemorrhage by comparing the clinical data, the treatment of intracerebral hemorrhage by neuroendoscopy and conventional drainage, and the differences in postoperative and prognosis of patients. The results of this study showed that compared with the routine trepanation and drainage group, the neuroendoscope combined with intraventricular lavage group had obvious advantages in the perioperative period (*p* < .05). It was suggested that neuroendoscopy combined with intraventricular lavage can effectively shorten the operative time and CSF drainage time and reduce the intracranial infection rate in patients with severe IVH. It is hypothesized that this may be related to the placement of the drainage tube under the neuroendoscope, which can place the drainage tube in an accurate position, so as to improve the drainage efficiency. In addition, the removal of hematoma and bloody CSF under the neuroendoscope has a good visual field and can promote the recirculation of CSF. The results of this study showed that the hematoma clearance rate in the neuroendoscopy combined with intraventricular lavage group was significantly higher than that in the routine trepanation and drainage group (*p* < .05). It was suggested that neuroendoscopy combined with intraventricular lavage can effectively improve the clearance rate of hematoma in patients with severe IVH. The results showed that the GCS score, the recurrence rate of hematoma and the good/excellent rate of ADL in the neuroendoscope combined with intraventricular lavage group were significantly better than those in the routine trepanation and drainage group (*p* < .05), indicating that the intervention of neuroendoscopy combined with intraventricular perfusion in severe IVH can effectively improve the GCS score, reduce the recurrence rate of hematoma, and improve the ability of daily living of patients. This result is similar to the results of Zhang et al. who found that more patients in the neuroendoscopy group recovered well after 2 months according to the GCS score in the follow‐up data of 22 cases of IVH treated by neuroendoscopy (Zhang et al., 2007). Xiong, Yang, Huang, Chao, and Liu (2012) retrospectively analyzed the clinical data of 32 cases of hypertensive IVH treated with neuroendoscopy. They found that the hematoma clearance rate was 85.4% in all the patients. The GCS score at 1 week postoperatively was significantly higher than that before operation. Ping et al. explored the efficacy and application value of endoscopic hematoma removal and extraventricular drainage (EVD) in the treatment of severe IVH. A retrospective analysis of 42 patients with IVH revealed that the hematoma clearance rate was significantly improved in patients using neuroendoscopy, and the postoperative GCS score was significantly higher than that in the EVD group (Song et al., 2018). The experimental data of this study are more sufficient, which can show that neuroendoscopy has certain advantages in clinical treatment.

The specific reasons are summarized as follows: (a) the surgical approach is selected in the relatively safe area of the brain, surgeon can look directly at the hematoma through neuroendoscope, hence the operation is more direct and minimally invasive. The position and direction of the drainage tube can be continuously adjusted in the ventricle to fully remove the hematoma according to the need. The excessive traction of brain tissue was placed and repeatedly perfused using gentamicin saline during the operation, and the residual blood clots in the ventricle can be completely removed in order to improve the clearance rate of hematoma and improve CSF obstruction and to prevent postoperative infection and hydrocephalus (El Damaty, Marx, Fleck, & Schroeder, 2017; Ramakrishna, Kim, Bly, Moe, & Ferreira, 2016). (b) Neuroendoscopy can provide a good and reliable light source and open field during operation, which is beneficial to surgical operation. If arterial bleeding occurs during brain operation, unipolar or bipolar attractor electrocoagulation can be used to stop bleeding, which could significantly reduce the rebleeding postoperatively. If venous bleeding occurs, cotton tablets and hemostatic yarn can be used to stop bleeding in time to effectively reduce the recurrence rate of hematoma. (c) Neuroendoscopy can accurately locate the hematoma and clearly distinguish the location of the hematoma and the normal structure around it, so that surgeon can quickly clear the ventricle and intracranial hematoma during the operation, avoid the blood vessels, and release bloody CSF. The reduction of compression and stimulation of the surrounding normal brain tissue is beneficial to reduce the intracranial pressure and increase the effect and safety of the operation, so as to improve the ability of daily life of the patients. However, based on the clinical experience, the author believes that the use of neuroendoscopy in patients with severe IVH still needs to be improved as follows: (a) the site of hematoma in neuroendoscopic surgery is in the brain. However, it is difficult to implement in patients with no obvious ventricle after placement of the drainage tube. The pressure of brain tissue in the abovementioned patients is higher during the operation, which limits the operation space of neuroendoscope, and the operation is not easy to succeed. (b) Neuroendoscopic surgery for patients with obvious cast of the fourth ventricle, a single removal equipment displays limited space on the screen for other hematomas. The hematoma of the fourth ventricle should be removed at the same time, and CSF circulation should be improved, which is beneficial to the prognosis of the patients. (c) The surgical instruments such as attractor and gun bipolar coagulation should be updated in time before neuroendoscopic surgery, which is helpful to the quick and safe operation of neuroendoscopic surgery.

Taken together, given that neuroendoscopy combined with intraventricular lavage can effectively improve the therapeutic effect of severe IVH in terms of hematoma clearance rate and GCS score and improve the prognosis of patients, it is considered as a safe approach and we recommend its wide application for clinical practice.

## CONFLICT OF INTEREST

The authors declare there is no conflict of interest.

## AUTHOR CONTRIBUTIONS

Hai‐Tao Ding and Quan‐Min Nie contributed to the conception and design of the study. All authors participated in the clinical practice, including diagnosis, treatment, consultation, and follow‐up of patients. Yao Han and De‐Ke Sun contributed to the acquisition of data. De‐Ke Sun and Hai‐Tao Ding contributed to the analysis of data. Hai‐Tao Ding wrote the manuscript. Quan‐Min Nie revised the manuscript. All authors approved the final version of the manuscript.

### Peer Review

The peer review history for this article is available at https://publons.com/publon/10.1002/brb3.1756.

## Data Availability

The datasets generated and analyzed during the current study are available from the corresponding author on reasonable request.
